# Effect of Strain Rate and Temperature on Tensile and Fracture Performance of AA2050-T84 Alloy

**DOI:** 10.3390/ma15041590

**Published:** 2022-02-20

**Authors:** Nagaraj Ekabote, Krishnaraja G. Kodancha, T. M. Yunus Khan, Irfan Anjum Badruddin

**Affiliations:** 1 School of Mechanical Engineering, KLE Technological University, Hubballi 580031, India; ekabotenagaraj@gmail.com; 2 Research Center for Advanced Materials Science (RCAMS), King Khalid University, 9004, Abha 61413, Saudi Arabia; yunus.tatagar@gmail.com; 3 Department of Mechanical Engineering, College of Engineering, King Khalid University, 394, Abha 61421, Saudi Arabia; magami.irfan@gmail.com

**Keywords:** AA2050-T84 alloy, strain rate effect, plane strain fracture toughness, temperature effect, strain rate effect, constraint effect

## Abstract

AA2050-T84 alloy is widely used in primary structures of modern transport aircraft. AA2050-T84 is established as a low-density aluminum alloy with improved Young’s modulus, less anisotropy, and temperature-dependent mechanical properties. During flights, loading rate and temperature variation in aircraft engine subsequent parts are commonly observed. The present work focuses on the effect of loading rate and temperature on tensile and fracture properties of the 50 mm thick (2-inch) AA2050-T84 alloy plate. Quasi-static strain rates of 0.01, 0.1, and 1 s^−1^ at −20 °C, 24 °C and 200 °C are considered. Tensile test results revealed the sensitivity of mechanical properties towards strain rate variations for considered temperatures. The key tensile properties, yield, and ultimate tensile stresses were positive strain rate dependent. However, Young’s modulus and elongation showed negative strain rate dependency. Experimental fracture toughness tests exhibited the lower Plane Strain Fracture Toughness (*K*_IC_) at −20 °C compared to 24 °C. Elastic numerical fracture analysis revealed that the crack driving and constraint parameters are positive strain rate dependent and maximum at −20 °C, if plotted and analyzed over the stress ratio. The current results concerning strain rates and temperatures will help in understanding the performance-related issues of AA2050-T84 alloy reported in aircraft applications.

## 1. Introduction

Modern aircraft predominantly use lightweight structures to improve the performance-to-weight ratio. The low-density Aluminum alloy is popular among aircraft structures owing to its durable mechanical properties and ease of manufacturability [1,2]. Composites pose tough competition to Aluminum alloys due to their tailor-made properties suited for specific applications. The unpredictable behavior of composites for change in temperature and time, restricted its usage to secondary and tertiary aircraft structures [3]. Currently, the modern transport aircraft primary structures are built by Al-Li alloys. The Lithium addition to aluminum with improved manufacturing methods resulted in the enhancement of specific strength and stiffness of the alloy [4]. However, the higher cost of Al-Li alloy restricted its usage to only aerospace industries.

The apprehensions related to diversifying mechanical properties of 1st and 2nd generation Al-Li alloys directed the complete withdrawal from aerospace applications [2]. Some noteworthy limitations were anisotropic behavior, cracking during manufacturing, and thermal instability-driven lower fracture toughness [2]. The skillful and sophisticated fabrication methods steered the evolution of 3rd generation Al-Li alloys. The spars and ribs of modern transport aircraft are fabricated by a 3rd generation Al-Li alloy, AA2050-T84 [1]. AA2050-T84 alloy exhibits exceptional tensile, fatigue, and fracture toughness behavior suited for damage tolerance property requirements of wing structures [1]. Notably, the anisotropic behavior and temperature-dependent property variations of AA2050-T84 alloy were also reported [5,6]. During flights, the fuel pressure at various altitudes of an aircraft, wing lift, and drag loads at different operating conditions may result in load rate and temperature variations on spars and ribs. The sensitivity of AA2050-T84 alloy to these load and temperature variations are essential to claim its suitability to modern transport aircraft wing parts. The following paragraphs discuss the strain rate and temperature effect on various ductile and brittle material properties reported in the literature.

Mirza et al. [7] have conducted tensile tests on mild steel and aluminum at various quasi-static (lower strain rates up to 1 s^−1^) strain rates. The results have shown negligible dependency of ductility on strain rate variations. Clausen et al. [8] have reported the negative strain rate sensitivity of AA5083–H116 through tensile tests for quasi-static strain rate variations. The strain rate dependency was related to dynamic strain aging at lower strain rates and temperatures, resulting in serrated stress-strain curves. Singh et al. [9] have reported increased flow stress with the rise in test temperature and strain rates on titanium alloys. The observed trend was attributed to the dynamic strain aging of the alloy. Through experiments and numerical analysis, Khan et al. [10] have investigated the influence of strain rate and temperature on Al2024-T351. The results inferred a strong temperature dependency and negligible strain rate effect on fracture strength of the Al2024-T351 alloy. Anderson et al. [11] have witnessed the sensitivity of DP 780 steel towards the strain rate variations. The tensile stress-strain response was steady and almost negligible concerning quasi-static strain rates. The alterations in failure surface morphology were noticed with changes in strain rates.

The experimental tensile results on DP590 and TRIP 780 steel by Roth et al. [12] have shown that ductility increases with loading speed. Rincon et al. [13] have studied the influence of temperature (between −90 °C to 270 °C) on tensile behavior of an as-cast A319 alloy and noticed the silicon dominant brittle fracturing regardless of temperature variation. Natesan et al. [14] have reported the variation in strain rate effect at different temperatures on the deformation behavior of A356-T7 cast aluminum alloys. The yield stress and strain hardening of Aluminum alloy 7075-W exhibited the positive load rate effect and negative temperature effect through plasticity experiments [15]. Hafley et al. [5] and Chemin et al. [6] have reported AA2050-T84 alloy tensile and fracture properties sensitivity to temperature variations. In summary, the material properties of various alloys of steel and aluminum generally exhibit reliance on strain rate and temperature and are noteworthy.

The dependency of fracture behavior on strain rate and temperature mainly alters the state of stress near the crack front. The state of stress variation at the crack front due to specimen type, geometry, and load type was defined by a term constraint. The constraint level at the crack component/structure drives the selection of standard test specimens for fracture toughness tests [16,17]. In Linear Elastic Fracture Mechanics (LEFM), the variation of the state of stress near the crack was measured by popular constraint parameters [18,19,20,21,22,23,24,25,26]. *T*_11_ and *T*_33_ are used to measure the in-plane and out-of-plane constraints in LEFM. The variations of *T*_11_ and *T*_33_ concerning specimen type, geometry, and load were well documented [16,18,19,20,21,22,23,24,25,26]. However, the strain rate effect at different temperatures on tensile and fracture behavior of the AA2050-T84 alloy is essential to claim its suitability to primary structures of the aircraft wing. Fracture toughness standard methods recommend the single value of fracture toughness for quasi-static load variations [27]. Notably, the above literature study shows the strong dependency of material properties on strain rate and temperature variations. Moreover, critical cracks were observed in AA2050-T84 made spars and ribs of Airbus-380 aircraft after a few flights [28]. The present literature findings demand a fracture study based on constraints near the crack of AA2050-T84 alloy at different strain rates and temperatures.

In the present work, AA2050-T84 alloy tensile behavior at different strain rates and temperatures are experimentally studied. Experimental fracture toughness tests are conducted using Compact Tension (C(T)) specimens at various temperatures. Furthermore, the effect of strain rate on fracture characterizing parameters in LEFM such as Stress Intensity Factor (*K*_I_), *T*_11,_ and *T*_33_ are analyzed numerically for different temperatures. Finally, the AA2050-T84 alloy tensile and fracture behavior dependency on strain rates and temperatures are compared and evaluated for compatibility for aircraft wing structures.

## 2. Material and Test Details

### 2.1. AA2050-T84 Alloy

This study uses a 50 mm thick (2-inch) AA2050-T84 alloy plate to extract the test specimens. The chemical composition of AA2050-T84 alloy in wt% as obtained from the supplier is shown in Table 1. Copper is used in AA2050-T84 to provide high strength, suited for aircraft applications [29]. The Lithium addition is (<1%) restricted to balance between density reduction and increase in Young’s modulus of the alloy [3,29].

### 2.2. Tensile and Fracture Toughness Test

**Figure 1:**
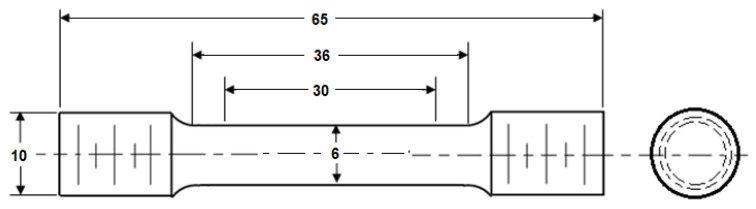
Tensile test specimen. All dimensions are in mm.

ASTM E8/E8M-21, the standard test method for tension testing of metallic materials [30], was used for the tensile specimen preparation and testing of AA2050-T84 alloy. Round specimens were extracted in the rolling (along the length of the plate) direction of the AA2050-T84 plate. Figure 1 shows the tensile test specimen dimensions (in mm) used in this study. The main dimensions of the specimen are, gauge diameter (*D*_0_ = 6 mm), gauge length (*L*_0_ = 30 mm), and overall specimen length (*L* = 65 mm). The tensile specimens were designed, keeping the *L_*0*_/D_*0*_* ratio to 5.

**Figure 2:**
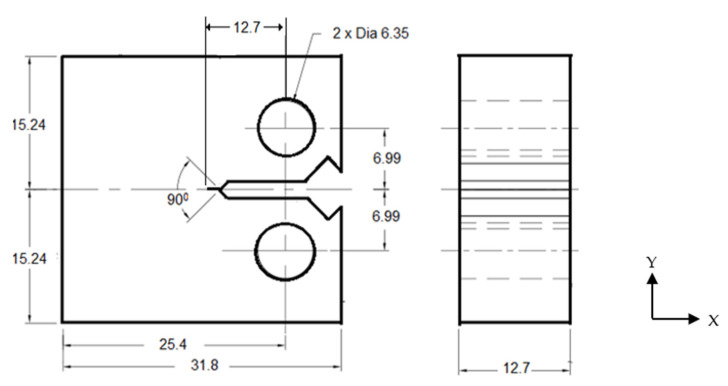
Compact Tension (C(T)) Specimen. All dimensions are in mm.

ASTM E399-20a, the standard test method for linear-elastic plane strain fracture toughness of metallic materials [27], was used. The commonly used fracture test specimen for primary aircraft structures is the compact tension (C(T)) specimen shown in Figure 2. The standard dimensions (in mm) are specimen width (*W* = 25.4 mm), specimen height (*H* = 2*W*), and specimen thickness (*B*) = crack length (*a*) = 0.5W. The C(T) specimen is extracted, ensuring the crack length in the rolling direction and load application in the transverse directions of the plate.

Flight durations and operations cause the temperature variations of the wing parts. Furthermore, the effect of these variations depends on alloy type and its ductile to brittle transition temperature [31]. However, temperature variations will be high near the aircraft engine (wing components), and the experienced load rates are dynamic. In the present study, the quasi-static strain rates considered in the tensile tests were 0.01, 0.1, and 1 s^−1^. The temperatures considered were −20 °C (Sub-zero temperature), 24 °C (Room temperature), and 200 °C (High temperature) [32]. In the tensile and fracture toughness tests, the low-temperature chamber with liquid nitrogen and a high-temperature furnace with forced convection heating was used to maintain the sub-zero and high temperatures. The Servo Electric Universal Testing Machine (UTM) (BISS, Bangalore, India) with 50 kN capacity was used for tensile and fracture toughness tests. In tensile testing, the applied load and deformations were recorded continuously through the load cell and extensometer, respectively. However, along with these, Crack Opening Displacement (COD) gauge (BISS, Bangalore, India) was used to record the relative displacement of two knife edges of the C(T) specimen in the fracture toughness test. These data were further processed to extract the tensile properties and fracture toughness of the AA2050-T84 alloy as per standards [27,30].

### 2.3. Finite Element Analysis

The crack driving forces and constraints of the C(T) specimen were investigated at different load rates and temperatures using 3D linear elastic finite element analysis (FEA). Half-symmetry is modeled and analyzed using Abaqus (6.14, 2014, Dassault Systemes Simulia Corp., Providence, RI, USA). The Poisson’s ratio (*ʋ*) and Young’s modulus (*E*) obtained from experimentally conducted tensile results at different strain rates and temperatures are used for linear elastic fracture analysis. The output parameters viz. stress intensity factor (*K*_I_) and constraint parameter (*T*_11_) were extracted using the counter-integral method mentioned in Abaqus post-processor [33]. *T*_33_ is calculated by using Equation (1). In Equation (1), *ε*_33_ is a strain in the *z*-direction (thickness direction) extracted along the crack front. The material property input and the *K*_I_ extraction details were adopted as similar to the work of [22,33].

(1) $$T_{33}=E\varepsilon_{33}+ʋT_{11}$$

**Figure 3:**
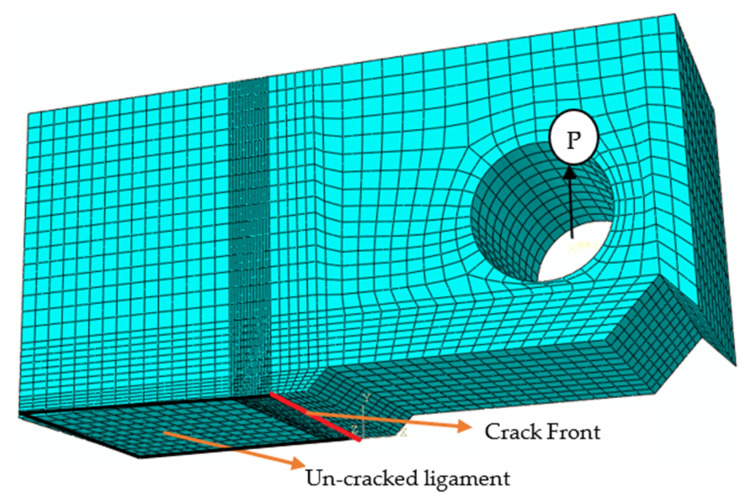
C(T) Meshed Model with Boundary conditions.

Half symmetry C(T) meshed model with supports and loading is shown in Figure 3. 20-noded hexahedral elements with reduced integration were used for the meshing. A fine mesh near the crack front was used to encapsulate the crack characteristics effectively. Singularity at the crack front was emulated by shifting the mid-side nodes of crack surrounding elements towards the crack front. The crack edge (crack front) surrounded by these nodes is defined as contour integral. The output parameters are calculated along the user-defined contour integrals (in the present analysis, it is 10 contours). The detailed procedure to define the crack front and contour integrals to obtain crack driving parameters is available in the Abaqus manual [33]. Y-symmetry was imposed along the ligament (the uncracked portion in the crack plane), and the tensile load was applied through the hole to simulate Mode-I.

## 3. Results and Discussions

The following sections discuss the experimental and numerical analysis of the AA2050-T84 alloy.

### 3.1. Experimental Analysis

#### 3.1.1. Tensile Test Analysis

**Figure 4:**
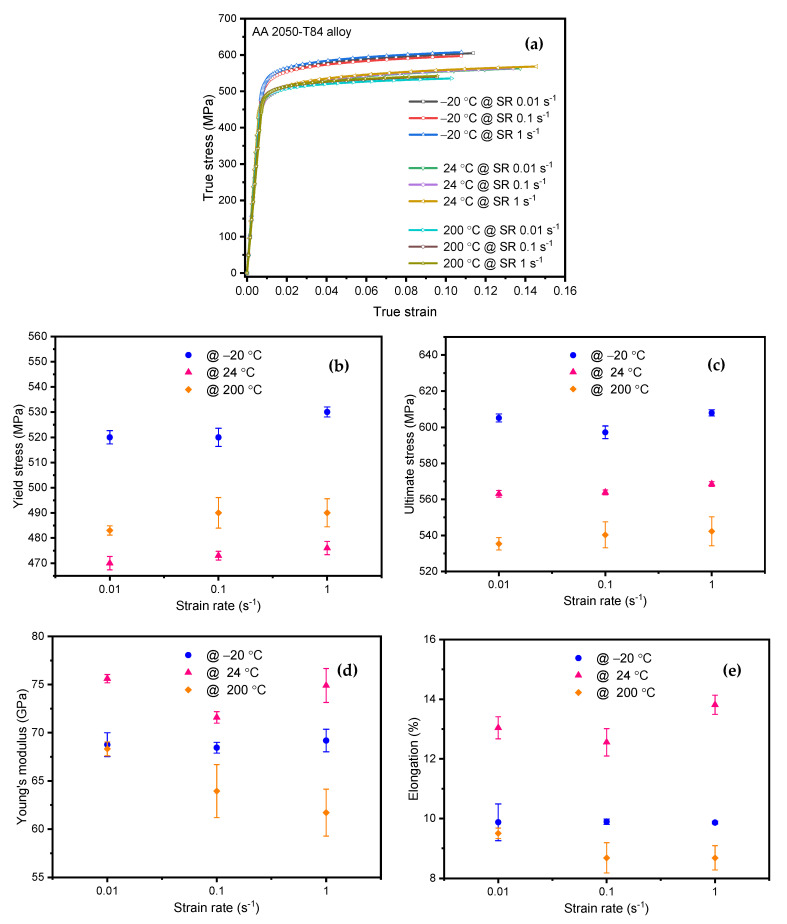
Strain rate and Temperature effect on: (**a**) True stress-strain curves (**b**) Yield stress; (**c**) Ultimate stress; (**d**) Young’s modulus; (**e**) elongation.

The experimental tensile tests were conducted at varying temperatures and strain rates. A total of 27 tensile tests were conducted, comprising 3 experiments for each strain rate per temperature. The stress-strain curves for different strain rates and temperatures are shown in Figure 4a. The corresponding variation of average tensile properties viz. yield stress (*σ*_ys_), ultimate tensile stress (*σ*_ut_), Young’s modulus (*E*), and % elongation (% et) extracted from the stress-strain curves along with error bars are presented in Figure 4b–e. The positive strain rate dependency was observed for tensile yield stress for the temperatures considered in the study, as shown in Figure 4b. The highest yield stress variation of 2% between 0.01 s^−1^ to 0.1 s^−1^ and 0.1 s^−1^ to 1 s^−1^ was observed at −20 °C. However, the lowest yield stress variation between successive strain rates, around 0.5%, was noticed at 24 °C. It was observed that the yield stress decreased from −20 °C to room temperature and further increased slightly at 200 °C, indicating the V-shaped behavior for temperature variation.

Similarly, ultimate tensile stress exhibited the positive strain rate dependency at various temperatures, as shown in Figure 4c. However, strain rate has minimal effect on ultimate tensile stress as the difference observed between successive strain rates for all temperatures is less than 1%. The ultimate tensile stress is inversely proportional to the temperature for all the strain rates and is in line with the observations of Hafley et al. [5] and Chemin et al. [6]

Figure 4d, shows the variation of Young’s modulus at various strain rates and temperatures. Young’s modulus showed negative strain rate sensitivity at room and higher temperatures. However, the strain rate effect on Young’s modulus was negligible (around 1%) at −20 °C. The maximum Young’s modulus difference of about 10% was observed at 200 °C between strain rates 0.01 and 1 s^−1^. Furthermore, Young’s modulus difference was around 5% at room temperature for successive strain rate variations. This reveals that the strain rate sensitivity towards Young’s modulus was in the decreasing order of temperatures 200 °C:24 °C:−20 °C. At 200 °C. Young’s modulus values were minimal and almost similar in values at −20 °C.

Overall, a reduction between 6% and 10% is noticed in Young’s modulus for the temperatures studied. The exact thickness of the plate with room temperature and −54 °C has been studied by Chemin et al. [6], revealing the same trend with a 2.5% reduction in Young’s modulus. With a 100 mm (4-inch) plate, Hafley et al. [5] noticed a 9–11% reduction in Young’s modulus when studied at different locations for room and −196 °C temperature.

Strong interatomic bonding between the atoms at room temperature may be the probable reason for the highest value of Young’s modulus. Farraro and McLellan [34] have reported that the lower values of Young’s modulus at elevated temperature indicate weakened interatomic bonding between the atoms.

Figure 4e shows the % elongation of AA2050-T84 alloy at different strain rates and temperatures. Negative strain rate dependency on % elongation as similar to Young’s modulus was observed for various temperatures. The maximum % elongation was noticed at room temperature, indicating higher ductility than other temperatures.

#### 3.1.2. Fracture Toughness Test Analysis

The fracture toughness tests were conducted as per ASTM E399-20a at different temperatures. ASTM E399-20a essentially elucidates the procedure of obtaining the single value, Plane Strain Fracture Toughness (*K*_IC_), for metallic materials under quasi-static strain rates. The C(T) specimen was fatigue pre-cracked to emulate the natural crack characteristics. The effect of loading and material properties (mainly yield stress) strongly influences fatigue crack growth [35]. The pre-cracking load details are shown in Table 2. The load ratio (*σ*_min_/*σ*_max_) was maintained at 0.1 to attain the *a/W* range in between 0.45 and 0.55.

**Figure 5:**
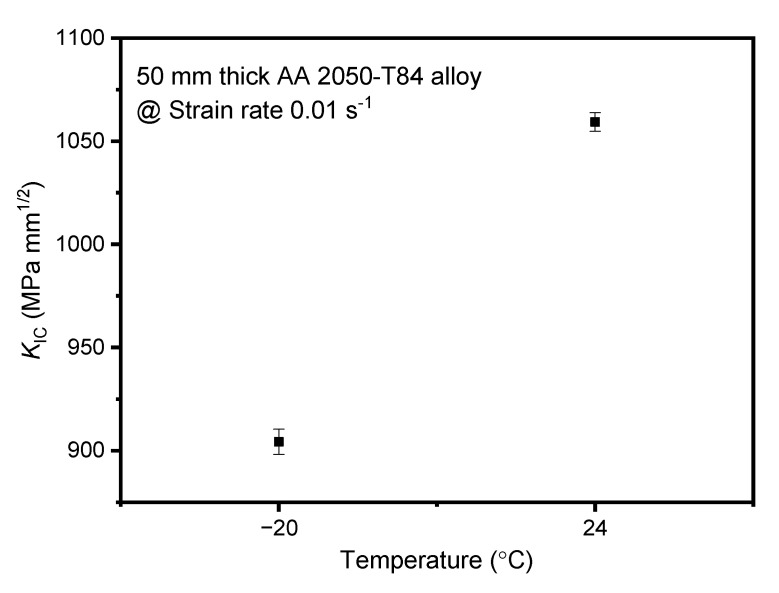
Plane Strain Fracture Toughness (*K*_IC_) at various temperatures.

The pre-cracked C(T) specimen was tested under Mode-I (opening mode) loading through tensile load application at the holes. A minimum of 3 successful fracture toughness tests was conducted at each temperature at the strain rate 0.01 s^−1^. The *K*_IC_ (average of 3 test samples) obtained from experiments for −20 °C and 24 °C are 904.28 and 1059.36 MPa mm^1/2^, respectively. The error bar for the *K*_IC_ is depicted in Figure 5.

At 200 °C, all the 3 test results were invalid as the ample crack deviation was observed from the crack plane. However, the *K*_IC_ has reduced by about 15% from room to sub-zero temperature. Similarly, in the work of Chemin et al. [6], a reduction of the order of magnitude 16% in *K*_IC_ was noticed in the rolling direction of the AA2050-T84 alloy plate from room to cryogenic (−56 °C) temperature. The decrease of *K*_IC_ at sub-zero temperatures of AA2050-T84 alloy can be attributed to surface hardening inside the grain and validated by the grain microstructure of the alloy [6,36]. In summary, the positive temperature dependency was witnessed for *K*_IC_ of AA2050-T84 alloy at strain rate 0.01 s^−1^. The economic limitations on conducting further fracture toughness tests at other strain rates impelled us to adopt the numerical analysis.

### 3.2. Linear Elastic Fracture Analysis

**Figure 6:**
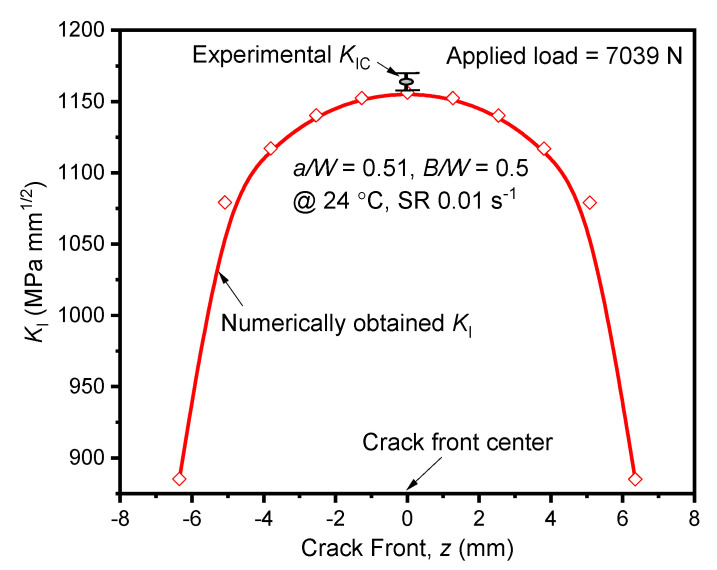
Stress intensity factor (*K*_I_) along the crack front at 24 °C.

ASTM E399-20a, to predict the *K*_IC_ of metallic materials, recommended the single toughness value for lower strain rate variations. However, the variation of tensile properties of AA2050-T84 alloy at different lower strain rates and temperatures was substantial. The elastic fracture analysis was carried out at varied strain rates and temperatures using the Abaqus software. The current numerical procedure was adopted from Kudari et al. [22] and Kavale et al. [37] The *K*_I_ values extracted through-thickness direction of the crack at 24 °C conditions, and experimental *K*_IC_ are shown in Figure 6. The experimental *K*_IC_ value was emulated through numerical fracture analysis with less than 1% error, as observed in Figure 6.

Similarly, at −20 °C, the validation of the numerical procedure was executed with less than 1% error. For all the numerical analysis, the center of the crack front was associated with the largest value of the crack characterizing parameter. Thus, the *K*_I_ values at the center of the specimen are used for analysis in further discussions.

#### 3.2.1. Effect of Strain Rate

**Figure 7:**
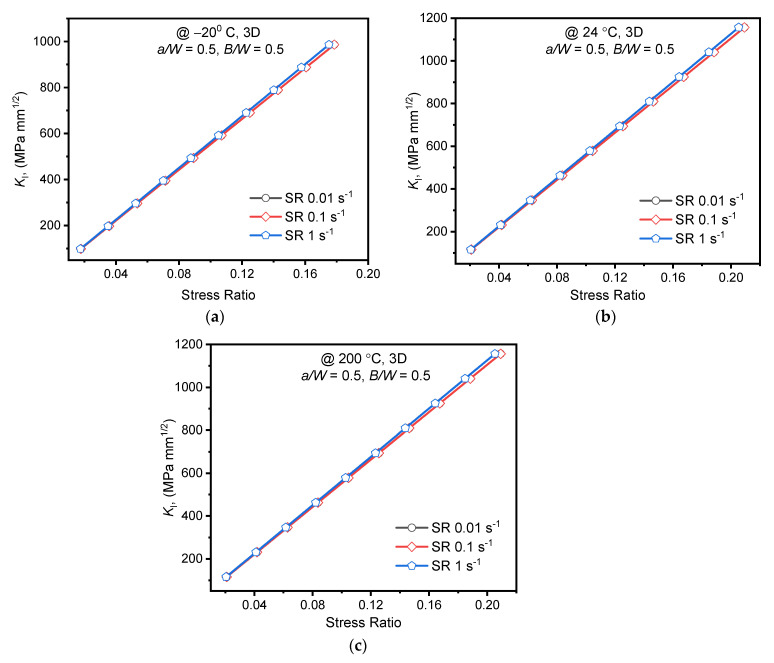
*K*_I_ at specimen thickness center obtained using FEA for different strain rates vs. Stress ratio (**a**) @ −20 °C; (**b**) 24 °C; (**c**) 200 °C.

In linear elastic fracture analysis, the experimental load associated with *K*_IC_ of the alloy is the applied load at respective temperatures. However, the *K*_IC_ at 200 °C was unavailable, and hence for the numerical analysis, the assumed load applied up to *K*_I_ = 1200 MPa mm^1/2^. The applied stress (*σ*_applied_) was determined using the relationship mentioned in Equation (2) [27]. The extracted values of *K*_I_ at the crack front center for various strain rates and temperatures are plotted against the stress ratio (*σ*_applied_*/σ*_ys_) as shown in Figure 7. Positive strain rate dependency of the *K*_I_ was observed for all temperatures considered in this study. At room temperature, a steady increase of 0.6% in *K*_I_ was witnessed with the rise in strain rate. However, for strain rate 1 s^−1^, the maximum of 1.88% increase in *K*_I_ was noticed at −20 °C. The strain rate sensitivity on *K*_I_ was found to be maximum at −20 °C and minimum at 24 °C, as the same trend was noticed for tensile yield stress values. The results of the *K*_I_ are in line with the yield stress variations of the alloy for all strain rates at different temperatures. The nominal variations of *K*_I_ (within the stress ratio of *K*_IC_) indicate the negligible dependency of fracture characterizing parameters on strain rates for AA2050-T84 alloy. Moreover, ASTM E399-20a recommendation to use the single value of *K*_IC_ for quasi-static strain rates seems to be justifying as the difference in numerical *K*_I_ values was minimal.

(2) $$K_{I}=\frac{P_{\mathrm{applied}}}{B\sqrt{W}}\;f\left(\frac{a}{W}\right)$$

**Figure 8:**
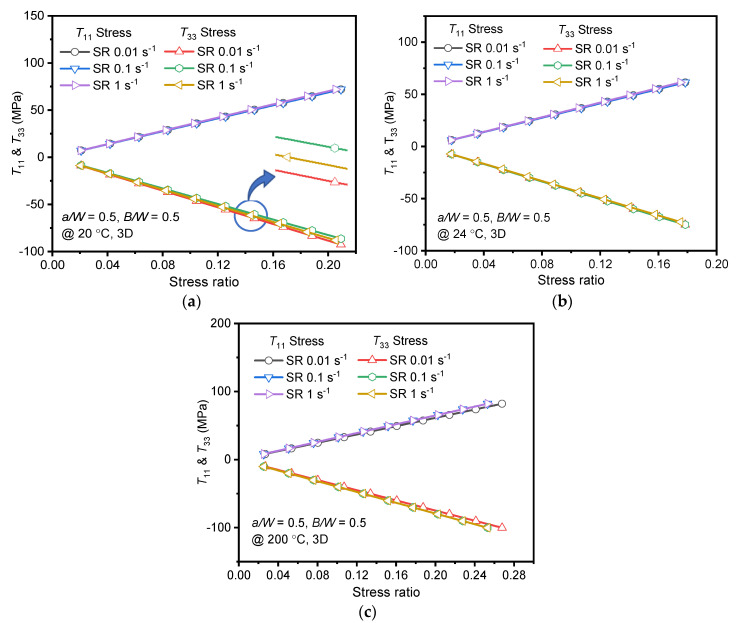
*T*_11_ and *T*_33_ at specimen thickness center obtained using FEA for different strain rates vs. Stress ratio (**a**) @ −20 °C; (**b**) 24 °C; (**c**) 200 °C.

Further, the effect of strain rate on crack tip/front constraints has been evaluated through *T*_11_ and *T*_33_. The values of *T*_11_ and *T*_33_ are found to vary along the thickness similar to *K*_I_ variation and maximum being at the center of the specimen. One can infer that the crack-front constraint is high at the center than at the surface; the material may fail at the center than on surface or shows instability at the center of the specimen. As the constraint parameters do not have a unique value for the specimen thickness, maximum values at the center of the specimen are considered for further analysis. *T*_11_ and *T*_33_ variations for different strain rates within the purview of *K*_IC_ (or stress ratio) of the alloy at respective temperatures are plotted in Figure 8. The nature of variation was identical at all strain rates for both constraint parameters. However, the increase in stress ratio resulted in positive *T*_11_ and negative *T*_33_ values at all strain rates and temperatures. It is clear from Figure 8, that the applied stress was directly proportional to *T*_11_ and inversely proportional to *T*_33_. The negative strain along the thickness resulted in the negative *T*_33_ [20]. This is in close agreement with the findings of Kudari et al. [22] for IF steel C(T) specimen.

At 24 °C and 200 °C, both constraint parameters were unaffected (very marginal difference) by the strain rate variation, as observed in Figure 8b,c. However, *T*_33_ variations between the strain rates at −20 °C were relatively substantial. At −20 °C, between the strain rates, a difference of 2.4% was found for *T*_33_. *T*_33_ variation depended on Poisson’s ratio and Young’s modulus (material property) of the alloy at different strain rates. Eventually, the crack driving and constraint parameters were less sensitive to strain rate variations.

Strain rate effect on *T*_11_ variation is negligible as in-plane constraint depends on specimen type, geometry, and loading type only. Furthermore, the variation of hydrostatic stress along the uncracked ligament is studied at different strain rates. It is observed that no variations are found at different strain rates. The negligible variation of *T*_11_ can also be attributed to the uniform state of stress at the crack front and minimal variation of yield stress (or stress ratio as depicted in the graph) between the strain rates. However, the *T*_33_ variation is quite measurable at different strain rates for −20 °C, as shown in Figure 8a, owing to the variations in material property (both Young’s modulus and yield stress). Positive *T*_11_ results in a lower plastic zone at the crack tip and influences the specimen’s unstable crack growth. Similarly, negative *T*_33_ results in loss of constraint at the crack tip.

Since the crack front plasticity is restricted in LEFM regime, the state of stress may be unaltered due to strain rate variations at identical temperatures. The current serrated stress-strain curves may affect the plasticity ahead of the crack front and can be accounted in Elastic-Plastic Fracture Mechanics (EPFM) regime.

#### 3.2.2. Effect of Temperature

**Figure 9:**
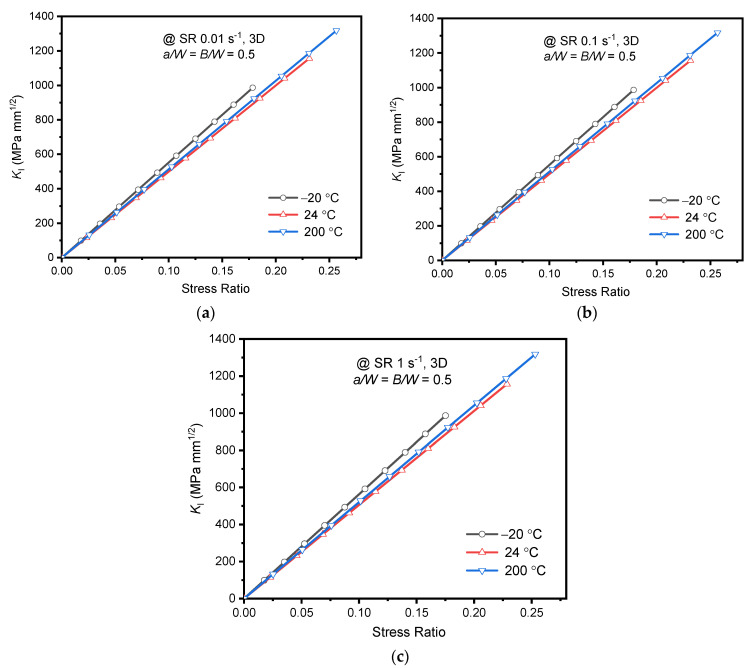
*K*_I_ at specimen thickness center obtained using FEA for different temperature vs. Stress ratio (**a**) SR 0.01 s^−1^; (**b**) SR 0.1 s^−1^; (**c**) SR 1 s^−1^.

The variation of *K*_I_ at different temperatures for quasi-static strain rates is plotted in Figure 9. The *K*_I_ variation is linearly increased with an increase in stress ratio as expected, and the nature of variation was the same for all temperatures considered. At the peak stress ratio, the difference between *K*_I_ at 200 °C and −20 °C is 5.1%, 5.16%, and 7.31% at strain rates 0.01, 0.1, and 1 s^−1^, respectively. However, the variation of *K*_I_ was minimum (around 4%) for the temperatures 24 °C and 200 °C at all strain rates. Notably, the temperature effect was highest at strain rate 1 s^−1^ and in the decreasing order of 1: 0.1: 0.01 s^−1^. The substantial variation of *K*_I_ at sub-zero temperatures indicates that for identical load conditions, the AA2050-T84 alloy is more prone to fracture failure than the other two temperatures as it possesses lower *K*_IC._

**Figure 10:**
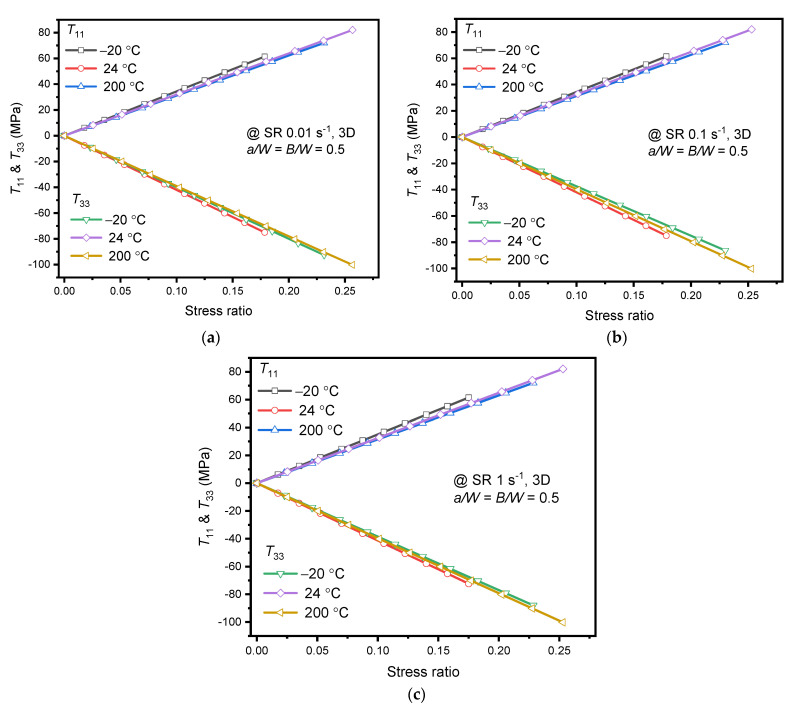
*T*_11_ and *T*_33_ at specimen thickness center obtained using FEA for different temperature vs. Stress ratio (**a**) SR 0.01 s^−1^; (**b**) SR 0.1 s^−1^; (**c**) SR 1 s^−1^.

Similarly, Figure 10 shows the variation of *T*_11_ and *T*_33_ at different temperatures for quasi-static strain rates. The variation of *T*_11_ and *T*_33_ were almost identical at 24 °C and 200 °C at quasi-static strain rates. At 200 °C, the *T*_11_ was less subtle and owed lower in-plane constraints than the −20 °C, and 24 °C. Chemin et al. [6] have related dislocations gathered along the grain boundaries, led to stress concentrations under loading and promoted the lower fracture toughness of the AA2050-T84 alloy at sub-zero (−56 °C) temperature. Similarly, in the current analysis, the sensitivity of fracture toughness and in-plane constraint against the stress ratio is highest at −20 °C. The sensitivity may be associated to crack front stress concentrations at grain boundaries at −20 °C and can be accounted through constraint parameters as shown in the current numerical analysis. However, the *T*_11_ and *T*_33_ variations seem identical for strain rates 0.01 and 1 s^−1^ through Figure 10a,c at −20 °C. Eventually, *T*_11_ and *T*_33_ in combination with *K*_I_ is maximum at 1 s^−1^ compared to 0.01 s^−1^. Thus, the highest constraint associated with numerically obtained *K*_I_ of the AA2050-T84 alloy at −20 °C is 1 s^−1^. This behavior also resulted in the lower *K*_IC_ at −20 °C than 24 °C.

In summary, the major constraint loss was observed for temperature variation compared to quasi-static strain rate variations. Moreover, at −20 °C, AA2050-T84 alloy possesses lower *K*_IC_ with the highest in-plane crack tip/front constraint compared to the other two temperatures. This behavior of the alloy makes it vulnerable to fracture failure in cryogenic (sub-zero) applications at a strain rate 1 s^−1^.

## 4. Conclusions

In the present study, the tensile and fracture behavior of the 50 mm thick (2-inch) AA2050-T84 plate was considered at various temperatures for quasi-static strain rates. Tensile tests revealed the sensitivity of mechanical properties towards the strain rates and temperatures. Positive strain rate dependency was observed for temperatures considered on yield stress and ultimate tensile stress of the alloy. A maximum of 2% increase in yield stress was noticed between strain rates at −20 °C. The lowest strain rate sensitivity of around 0.5% was witnessed at room temperature. Notably, the ultimate stress variation between the strain rates for temperatures was less than 1%. However, Young’s modulus and % elongation were negative strain rate dependent. The maximum decrease of Young’s modulus up to 10% was noticed at 200 °C. The minimum Young’s modulus variation was witnessed at −20 °C between the strain rates.

Temperature sensitivity towards tensile behavior of the AA2050-T84 alloy was noticed. The maximum yield stress variation of 10–11% was witnessed between room temperature and −20 °C. Notably, the yield stress increase was only up to 3% between room temperature and 200 °C. Similarly, for ultimate stress, the variation was up to 7.5% between room temperature and −20 °C. However, the reduction of Young’s modulus up to 18% was noticed between room temperature and 200 °C. This implies that yield and ultimate stress are quite substantial at −20 °C compared to other temperatures, making the AA2050-T84 alloy vulnerable at sub-zero temperatures. Moreover, an increase in strain rate prompts the decrease in % elongation, implying the brittle behavior of the alloy at higher strain rates.

The crack driving and constraint parameters are less sensitive to strain rate variations. However, at −20 °C, crack characterizing and constraint parameters to strain rate variations were moderately considerable. The temperature effect is highest at strain rate 1 s^−1^ and in the decreasing order of 1:0.1:0.01 s^−1^.

Overall, the AA2050-T84 alloy tensile and fracture performance obtained through experimental and numerical analyses exhibited the dependency on strain rates and temperatures. Hence, these mechanical properties of the alloy strongly influence the damage tolerance design of spars and wings of the aircraft. The authors believe that the current results concerning strain rates and temperatures will help in understanding the performance-related issues of AA2050-T84 alloy reported in aircraft applications.

## Figures and Tables

**Table 1:** Chemical composition of 50 mm thick (2-inch) AA2050-T84 alloy plate (wt%).

Cu	Mg	Mn	Zn	Fe	Ti	Si	Li	Zr	Ag	Al
3.743	0.369	0.372	0.025	0.045	0.040	0.039	0.798	0.087	0.398	Base

**Table 2:** Pre-cracking details of fracture toughness test.

Crack Length *a* (mm)	Crack Length/Width *a/W*	Maximum Stress *σ*_max_ (MPa)	Minimum Stress *σ*_min_ (MPa)	Mean Stress *σ*_mean_ (MPa)	Alternating Stress *σ*_average_ (MPa)
12.94	0.51	250	25	137.5	112.5

## Data Availability

This study didn’t report any data.
